# Methods matter: A primer on permanent and reversible interference techniques in animals for investigators of human neuropsychology

**DOI:** 10.1016/j.neuropsychologia.2017.09.019

**Published:** 2018-07-01

**Authors:** Andrew H. Bell, Janet H. Bultitude

**Affiliations:** a MRC Cognition and Brain Sciences Unit, University of Cambridge, Cambridge, UK; b Department of Experimental Psychology, University of Oxford, Oxford, UK; c Department of Psychology, University of Bath, Bath, UK; d Centre for Pain Research, University of Bath, Bath, UK; e The Centre for Functional Magnetic Resonance Imaging of the Brain, University of Oxford, Oxford, UK

**Keywords:** Animal models, TMS, tDCS, Lesion

## Abstract

The study of patients with brain lesions has contributed greatly to our understanding of the biological bases of human cognition, but this approach also has several unavoidable limitations. Research that uses animal models complements and extends human neuropsychology by addressing many of these limitations. In this review, we provide an overview of permanent and reversible animal lesion techniques for researchers of human neuropsychology, with the aim of highlighting how these methods provide a valuable adjunct to behavioural, neuroimaging, physiological, and clinical investigations in humans. Research in animals has provided important lessons about how the limitations of one or more techniques, or differences in their mechanism of action, has impacted upon the understanding of brain organisation and function. These cautionary tales highlight the importance of striving for a thorough understanding of how any intereference technique works (whether in animal or human), and for how to best use animal research to clarify the precise mechanisms underlying temporary lesion methods in humans.

## Highlights

• There are many ways to temporarily or permanently disrupt brain function in animals.
• Temporary vs. permanent disruption can sometimes lead to disparate results.
• Understanding the mechanisms of a given technique is vital to interpreting results.
• Here, we review current and emerging lesion techniques in animals.
• Animal models provide a valuable adjunct to neuropsychological studies in humans.

## 1 Introduction

To attribute a cognitive function to a particular brain region or network, several criteria must be met [see Parker and Newsome (1998) for discussion]. Typically, one might first establish a correlational relationship where brain activity is observed to change in predictable ways during changes in behaviour. To confirm a causal relationship, however, it is critical to *interfere* with the function of that brain region or network and establish that there is a measureable impact on behaviour.

One of the longest-established methods of determining a causal link between a given region or network and a cognitive function is through the study of patients with brain lesions. Classically, researchers infer such causal links when they can show that a lesion to a brain area impairs function A but not function B (a *dissociation*), and especially when they can also show that a lesion to a different brain area impairs function B but not function A [a *double dissociation* Teuber (1955)]. More recently, advances in neuroimaging techniques have improved our ability to map the precise boundaries of lesions, and new analysis techniques such as voxel-based lesion-symptom mapping (Bates et al., 2003) have enhanced our ability to link behavioural deficits with underlying damage (see other papers in this issue). Thus, the fundamental approach of examining lesions in human patients remains one of the most valuable tools for understanding brain function.

And yet, despite the undeniable contributions of patient studies to our understanding of cognition and brain function, they are nonetheless subject to some critical and unavoidable limitations. Conducting studies in animals, while controversial, addresses most of these limitations and thus provides a valuable adjunct to behavioural, neuroimaging, physiological, and clinical investigations in humans.

The purpose of this review is to help bridge the gap between these two approaches. In this review, we will:

• Summarise some of the key limitations of human lesion studies;
• Describe some of the current and emerging techniques for inducing lesions in animals. For the purposes of this review, we primarily focus on those techniques that are currently in common use with non-human primates because they are the animal model of choice for studying higher-order cognitive functions;
• Discuss limitations of animal lesion techniques, including instances where different lesion techniques have yielded different results, thus highlighting the importance of considering methodology when making conclusions; and
• Briefly comment on potential ways in which animal models can be used to improve our understanding and effective use of reversible techniques in humans.

We do not seek to provide a comprehensive list of all the techniques currently used in non-human species. Rather, we will provide a broad introduction to some of the underlying themes upon which these techniques are based. In doing so, we aim to illustrate how animal models serve to *complement,* not replace, human neuropsychology by addressing many of the limitations for human studies. We will demonstrate how animal models can *extend* human lesion studies by offering new tools, such as genetic approaches, that can tap into the mechanisms that underlie cognitive function in ways that are not possible in humans. Finally, we will highlight important insights from the animal literature when comparing the effects of temporary versus permanent lesions in humans.

## 2 Key limitations of human lesion studies

For almost 200 years, scientists and clinicians have carefully examined the behavioural deficits of brain-lesioned individuals or groups of patients to infer the function of the damaged brain area. Although the field of phrenology seems laughable today, its founder, Franz Josef Gall, based some of his localisation decisions on examinations of brain damaged individuals (Gall, 1835). Most notably, such patient-based observations led him to ascribe the ‘word memory’ area to an anterior frontal lobe region that is very near to the area identified by Paul Broca many years later (Broca, 1861; Brown, 1992). Other classic studies include Carl Wernicke's observations on language, John Hughlings Jackson on motor function, and John Harlow on executive function (Critchley and Critchley, 1998; Damasio et al., 1994; Gross, 1999; Harlow, 1848; O'Driscoll and Leach, 1998). Yet, as with any scientific method, human neuropsychology has some limitations for making inferences about cognitive function.

The main limitations are (see also Humphreys and Price, 2001):

• **Location:** Not only is every person's brain unique, researchers have no control over where the lesion occurs or how large an area it covers. Lesions are most typically caused by trauma or stroke. While lesions caused by trauma (e.g., gunshot wounds, blunt force trauma) can theoretically be located anywhere in the brain, lesions caused by stroke are, by definition, dependent upon the underlying vasculature of the brain. This means some brain regions are more likely to be affected than others (Corbetta et al., 2015, Kang et al., 2003, Wessels et al., 2006). For example, strokes will more often involve the middle and not the posterior cerebral artery, meaning that posterior cortex is affected relatively infrequently. Similarly, areas supplied by more than one cerebral artery will also rarely suffer ischemia. From a research perspective, this means that lesions almost never obey cytoarchitectonic borders or functional distinctions that allow researchers to address specific hypotheses about particular areas. It also means that it is highly unlikely that the area of scientific interest will be the only area affected in that patient. Indeed, some of the most influential neuropsychological cases in the scientific literature are thus defined due to the rare location and/or unusual focality of their lesion [e.g., Patient DF, who suffered extensive damage to the lateral occipital complex following carbon monoxide poisoning (Goodale et al., 1994); or Patient TN, who suffered two successive strokes resulting in near-complete bilateral damage to the occipital cortex (de Gelder et al., 2008)].
• **Patient experience:** With patient studies, we have no control over the health and life experience of the participant. Stroke patients are generally older and can have additional co-morbidities. Similarly, altered function in patients who have undergone resections to treat epilepsy could be due to either the surgical lesion, or the neurodegenerative consequences of recurrent seizures and/or associated head injury. This will lead to confounding variables that cannot be completely accounted for in the control group, or difficulty in interpreting brain changes that occur because of the lesion.
• **Time:** Although it is theoretically possible to test patients within the first several days after the initial trauma, these opportunities can be limited by patient drowsiness, the natural and understandable priority for the patient to spend time with visiting friends and family, and/or other injuries sustained by the patient (e.g., if a stroke led to a fall, or there is a brain injury associated with a car accident). So, for both compassionate and logistical reasons, patients are typically seen days, weeks, or even years after the injury; raising concerns about post-lesion reorganisation and/or compensation that might obscure the true function of a given area.

In short, there is an inherent confound in using a *permanently damaged brain* to understand the function of an *intact healthy nervous system.* By contrast, animal models offer the opportunity to study the neural bases of behaviour without many of these limitations. Animal models allow for substantially more control over *where* a lesion is located; potentially with exquisite control over the size/boundaries of the lesion (see below). Animal models also provide control over *when* the lesion takes place (e.g., before or after a given task is learned or knowledge is acquired); and *how soon after* the lesion and *how often* the subject is tested on the relevant cognitive tasks. The ability to test and re-test the subject grants greater statistical reliability and offers the opportunity to further assess recovery of function over an extended timeframe.

Until relatively recently, perhaps the most significant advantage of animal models over human studies was that it was the only way in which it was possible to study the effects of *reversible* lesions. Although reversible lesions do not completely eliminate the possibility for reorganisation, nor do they control for potential off-target effects (e.g., Otchy et al., 2015; see below), they nonetheless provide an opportunity to examine the *immediate* consequences of removing a specific brain region on behaviour. This particular advantage of animal models over human studies might be closing, thanks to the development of ‘reversible’ or ‘virtual’ lesion techniques such as transcranial magnetic stimulation (TMS) and transcranial direct current stimulation (tDCS) that can temporarily alter brain function in humans (see other papers in this issue). However, these new methods can also create new problems in that the effects can be subtle, the location of stimulation can be uncertain, the mechanisms of action are unclear, and the techniques are currently limited to cortical areas located on the dorsal and lateral surfaces (and not subcortical structures, or cortical structures located on the ventral or medial surfaces).

Perhaps because of these limitations, the findings from temporary inactivation versus permanent lesion studies in humans do not always correspond. For example, Van der Stigchel and his colleagues have shown that oculomotor inhibition is impaired in patients with permanent lesions to the frontal eye fields (Van der Stigchel et al., 2012), but enhanced by temporary inactivation of the same area using TMS (Bosch et al., 2013). Although techniques that temporarily inactivate brain tissue in humans could bypass some of the limitations of permanent lesions, they also raise new questions.

Thus, we have new opportunities – and new challenges. Techniques for inducing reversible lesions in animals have been around for much longer than for humans, and over this time researchers have identified several instances of divergence between results obtained with permanent lesions and those obtained with reversible lesions. These discrepancies were not just due to the presence of reorganisation, but also to methodological differences between different techniques that led to differences in the lesion substructure (such as whether they affected fibres of passage or not). This serves as an important reminder – a cautionary tale – for investigators seeking to use reversible lesion techniques in humans: one cannot necessarily expect the results from different methods to align, and for this reason it is important to have a thorough understanding of precisely what a given technique is doing before drawing conclusions from it. And so, we provide an overview of some of the techniques currently in use in animals with the aim of providing a different perspective for researchers looking to use temporary lesion techniques in humans. For this special issue, we focus on those techniques that are commonly used in rhesus macaques. We have chosen to focus on rhesus macaques as they are the most commonly used non-human primate species for studying higher-order cognition due to their having similar brain organisation to humans (see Preuss, 1995; Wise, 2008), and their ability to perform highly complex cognitive tasks most like those used in human neuropsychological research (e.g., the Wisconsin Card Sorting Task).

## 3 Lesion techniques in animals

Animal researchers have a wide variety of techniques available to them to permanently lesion or temporarily inactivate a region (see Table 1). The choice of technique comes down to a set of factors determined by the experiment and hypotheses to be tested. These include:

• **Size:** Does the lesion need to cover an entire cortical area, or only a small targeted region?
• **Location:** Is the intended lesion targeting areas of the brain that are more easily accessed (e.g., prefrontal cortex), or a more difficult-to-access brain area (e.g., subcortical areas, or cortical areas on the ventral or medial surface)?
• **Duration:** Does the lesion need to be permanent, or reversible? If the latter, how long should the effects of the temporary lesion last?
• **Tissue type and specificity:** Is it necessary to spare fibres of passage? Should the lesion selectively target grey versus white matter? Is it desirable to target a particular cell type (e.g., pyramidal neurons, interneurons, glia)?
• **Known mechanism of action:** Is it necessary to the hypothesis that the underlying method of disrupting neuronal function is clearly understood (e.g., aspiration versus TMS)?

**Table 1:** Permanent and reversible lesion techniques in animal and human research.

**Technique**	**Durations**	**Tissue Specificity**	**Relative Size**	**Spare Fibres?**
**ANIMAL**				
**Permanent**				
Aspiration	Permanent	Non-specific	Unlimited	No
Excitotoxic	Permanent	Specific	Small-Medium	Yes
**Reversible**				
Pharmacological Injections	Minutes to hours	Specific	Small	Yes
Cryogenic	Hours	Non-specific	Large	No
Genetic	Milliseconds to hours	Specific	Small	Yes
TMS	Millseconds to minutes	Non-specific	Small	No
FUS	Minutes	Non-specific	Small-Medium	No
**HUMAN**				
**Permanent**				
Lesion due to Stroke	Permanent	Non-specific	Variable	No
Lesion due to Trauma	Permanent	Non-specific	Variable	No
**Temporary**				
Wada test (Wada, 1949)	Minutes	Non-specific	Large (whole hemisphere)	No
TMS	Millseconds to minutes	Non-specific	Small	No
tDCS	Millseconds to minutes	Non-specific	Small to medium	No

FUS = Focused Ultra-Sound; tDCS = Transcranial Direct Current Stimulation; TMS = Transcranial Magnetic Stimulation.

### 3.1 Permanent techniques

#### 3.1.1 Aspiration

Creating permanent lesions through aspiration or excision is perhaps the oldest documented lesion technique, beginning with the work of researchers like Jean Pierre Flourens in the 1820's (see Pearce, 2009). While the surgical techniques and survival rates have improved dramatically over the past 200 years, the basic premise remains the same, and indeed aspiration remains an important part of the neurosurgical approach for excising tumours and epileptic foci in humans. Creating this type of lesion involves performing a craniotomy to remove a section of skull, exposing the brain by retracting the dura, and removing brain tissue using cautery and suction or excision. This approach has two distinct advantages over other methods of creating permanent lesions. The first is that it allows for *visually-*guided removal of one or more specific brain regions. Target regions are most often localised through visual landmarks such as sulci and vasculature. These have less inter-individual variability in the macaque brain as compared to the human brain, although it is important to note that functional boundaries can still deviate substantially between subjects. The second advantage of the aspiration technique is that it is the only commonly-used technique for creating permanent lesions that allows for the near-complete and confirmed removal of large areas of cortex, which might be necessary if an area does not feature a clear topographical organisation.

Creating lesions through aspiration also has several disadvantages. First, this approach requires that the targeted region be visible (or at minimum, surgically accessible) and, as such, places some constraints on potential targets. Aspiration has been used to target several more difficult-to-access areas, such as those found on the orbitofrontal (e.g., Bachevalier and Mishkin, 1986; Baxter et al., 2007; Meunier et al., 1997; Simmons et al., 2010) or ventral temporal (e.g., Buffalo et al., 1999; Eacott et al., 1994; Gaffan et al., 2002; Meunier et al., 1993a) surfaces, but cannot be used to target many subcortical areas (e.g., amygdala) without affecting neighbouring tissue (see below, Mishkin, 1978). Second, and perhaps most disadvantageous is that it is difficult to preserve all neighbouring tissue and/or fibres of passage using this technique. While clearly visible on an MRI or a fixed brain ex-vivo, the contrast between white and grey matter when viewed with the naked eye is much less pronounced and so the surgeon must strike a balance between ensuring removal of the target tissue and minimal disruption to white matter pathways.

#### 3.1.2 Excitotoxic lesions

Excitotoxic lesions are permanent lesions that have been created through the injection of a neurotoxin, such as ibotenic acid, which acts by overstimulating NMDA-receptors leading to an accumulation of glutamate and calcium, and ultimately cell death.

The main advantages of this approach directly address the disadvantages of aspiration lesions, which are that excitotoxic lesions do not necessarily require visual access to the tissue and thus can be used to lesion almost any part of the brain, including deep subcortical structures (e.g., Hamada and DeLong, 1992). Also, because these lesions rely on excessive neuronal responses to cause cell death, they can be tailored to target only neurons or even specific neurotransmitters, and thus spare fibres of passage. As we will explain later in this review, this distinction can have critical effects on the interpretation of findings.

However, with this flexibility comes some disadvantages. Most notably, it can be difficult to directly assess the spread and efficacy of the neurotoxic drug. This means it can be difficult to ensure the precise extent of the lesion.

Both aspiration and excitotoxic methods require a period of post-operative recovery before the animal can be tested. This period ranges from a few days to a few weeks depending on the brain area, lesion method, and nature of the research.

### 3.2 Reversible techniques

The number of options for temporarily inactivating an area in animals has expanded significantly over the past 10–15 years. While reversible, most of the techniques discussed below are to some degree invasive and therefore their use is restricted to animals.

#### 3.2.1 Pharmacological interventions

Like the approach used for the creation of excitotoxic lesions, this method involves injecting a compound (in this case, a non-neurotoxic compound) into a specific brain region to temporarily reduce neurotransmission. Typically, small volumes of drugs, such as the GABA-agonists muscimol and tetrahydroisoxazolo pyridine (THIP), are injected via a microsyringe directly into the brain region of interest. GABA-agonists will increase synaptic transmission of neurons with GABA-receptors, most notably inhibitory interneurons (Mann-Metzer and Yarom, 2002), thus leading to a decrease in the firing of output neurons in that area (Krogsgaard-Larsen et al., 1977). As with excitotoxic lesions, virtually any brain region can be targeted and this approach can be tailored to spare fibres of passage. Such drugs typically take approximately 10–20 min to take full effect, and wear off after a few hours (although lingering effects of the drug can be observed as long as 24 h post-injection).

Like excitotoxic lesions, however, it can be difficult to assess the spread and efficacy of the drug. Some researchers have begun to bind the drug to an MR-visible compound to confirm disposition of the drug (Wilke et al., 2010) but this approach only reveals location and does not confirm that the drug has successfully taken effect. Therefore, while this approach can be highly effective in temporarily inactivating small regions of the brain to produce specific effects (e.g., saccade dysmetria, Dias and Segraves, 1999; alterations in choice behaviour, Afraz et al., 2015; Gouvêa et al., 2015; etc.), it is potentially less reliable as compared to other techniques in producing robust wide-spread effects that require large-scale inactivation of tissues (e.g. complete hemianopia).

#### 3.2.2 Cryogenic Inactivation

Another method of shutting down activity in the brain temporarily is through the implantation of cryogenic probes or ‘loops’ (see Lomber et al., 1999; Payne and Lomber, 1999 for reviews). This approach involves surgically implanting a small loop of metal or plastic adjacent to the cortical surface, and then feeding into this loop a fluid that cools the tissue to the point where neuronal activity is reduced. It is possible to alter the degree of cooling and thus the period of inactivation by altering the properties of the loop and fluid. This approach is particularly well suited for inactivating larger regions of cortex (e.g., Peel et al., 2014). It does, however, require direct access to the tissue so, like aspiration methods, is not well-suited for deep and subcortical structures.

#### 3.2.3 Genetic-based approaches

A recent and exciting development in rodent models that is making its way to primate research (Berdyyeva and Reynolds, 2009, Cavanaugh et al., 2012, Gerits et al., 2012, Han et al., 2011, Han et al., 2009, Jazayeri et al., 2012) is one that combines genetic manipulation with either light-based or drug-based 'activators'. At present, the two most common genetic-based approaches used in non-human primates are known as ‘optogenetics’ (Deisseroth, 2010, Kim et al., 2017) and ‘chemogenetics’ – specifically DREADDS (‘Designer Receptors exclusively Activated by Designer Drugs’; Roth, 2016; Urban and Roth, 2015). Broadly speaking, these techniques use viral vectors to make cells express specific proteins. These proteins are then selectively triggered to alter cell function. For example, in the case of optogenetics, one experiment might involve making neurons express a transmembrane receptor (e.g., channelrhodopsin) that, when activated by a specific wavelength of light, opens and causes that neuron to fire action potentials. In the case of DREADDS, similar receptors might be activated by a drug (e.g., clozapine).

Genetic-based approaches have immense potential for neuroscience (see Tye and Deisseroth, 2012). It is theoretically possible to target not just individual structures, but individual *cell types* (e.g., Klein et al., 2016; Stauffer et al., 2016) or *pathways* (e.g., Inoue et al., 2015; Kinoshita et al., 2012; Oguchi et al., 2015). They also allow for precise control over the timing of the lesion and therefore, compared to permanent lesion methods, reduce the potential for the results to be confounded by reorganisation.

The success of genetic-based approaches depends on several steps, including uptake of the viral vector, expression of the protein(s), and ability to activate those proteins. Each of these steps is inherently challenging, meaning that only a fraction of the target cell populations may be affected. Over the past several years, however, there has been an exponential growth in the number of studies that have successfully used genetic-based approaches in monkeys. It is likely that these techniques will soon become commonplace in primate neuroscience research.

#### 3.2.4 Transcranial Magnetic Stimulation (TMS)

TMS uses a magnetic inducer (coil) that is applied to the scalp to produce electrical currents in the underlying brain tissue. Brain activity can be enhanced or suppressed depending on the precise parameters of stimulation. In the case of suppression, the duration of the effect can be anywhere between milliseconds (after a single TMS pulse) to many minutes (after several repeated TMS pulses). This technique is non-invasive and is routinely used in humans. Several laboratories are also using TMS to alter brain activity in animal models. This has the advantage of being the most easily relatable to results obtained in humans, but, as in humans, TMS can only be used to target cortical regions next to the skull surface. Furthermore, this approach has one disadvantage that is specific to its use in rhesus macaques. Rhesus macaques, particularly the large males that most laboratories rely on, have significant musculature on either side of the skull. This musculature, which can be as thick as several centimetres, makes it difficult to target lateral brain regions with TMS due to the discomfort experienced by the subjects. The musculature could also affect the focality of the TMS due to the dispersing effects of the muscles, and the increased distance between the coil and the cortex. Therefore, most labs that have used TMS in macaques restrict their stimulation sites to regions on the top of the brain, far from the muscles (Gerits et al., 2011, Gu and Corneil, 2014, Mueller et al., 2014, Valero-Cabre et al., 2012).

#### 3.2.5 Focused Ultra-Sound (FUS)

In this method, a section of skull bone is removed and an ultrasonic beam is used to irradiate a target area (Deffieux et al., 2013, Tufail et al., 2010, Tufail et al., 2011, Yoo et al., 2011). Both enhancement and inhibition of activity in an area can be induced depending on the settings used. To inhibit an area, an irradiation period of a few seconds or minutes is used, which will reversibly suppress activity for about 7 min.

FUS is an exciting complement to TMS, because it can be applied to deep structures in the brain. It can also offer the possibility of reversibly suppressing white matter tracts, thus enabling investigation of the structure of brain networks in addition to localisation of function to specific brain areas.

### 3.3 The crossed-lesion disconnection technique

The crossed-lesion disconnection technique (Ettlinger, 1959, Ettlinger et al., 1968, Mishkin, 1966) is distinguished not by the means through which the lesions are induced, but by the way in which those lesions are applied to answer a specific question. Most cognitive functions are no longer thought to be implemented in single brain areas, but rather through complex and time-locked interactions between distributed networks of brain areas. Therefore, when a function is lost after a brain area is permanently or temporarily lesioned, this could be either because the function depends on the lesioned area, or because the function depends on another area that now lacks input that is normally provided via its connection with the first. One way to distinguish between these two possibilities is to compare the effects of lesioning a single brain area to the effects of removing the fibres that connect multiple brain areas within a given network. However, the complexity of the connections between brain regions makes this approach nearly impossible.

With the crossed-lesion disconnection technique, area A is lesioned in one hemisphere and area B is lesioned in the other. If a function operates through a network that involves both areas A and B, then typically no or minimal impairments are observed in this preparation because the function can be achieved through connections with the intact areas of the opposite hemispheres. However, severing the connections between the hemispheres prevents the intact brain regions of the two hemispheres from communicating. If a deficit is observed once the hemispheres are disconnected, this confirms that the function is dependent upon the network between A and B.

This method has been applied to several different domains in neuroscience, including object recognition, reward, learning, and memory (e.g., Barefoot et al., 2002; Browning et al., 2007; Easton and Gaffan, 2001; Easton et al., 2001, Easton et al., 2002; Eldridge et al., 2016; Izquierdo and Murray, 2010; Mitchell et al., 2007) and is an example of how animal lesions studies can help researchers go beyond the classic localisation approach to establish the architecture of brain networks.

## 4 Methods matter: cautionary tales

Above, we have summarised several established and emerging techniques to permanently or temporarily alter brain function in animals, with a focus on non-human primate models. In the next section, we review a few cases where the limitations of one or more technique(s) and/or differences in their mechanism of action have impacted upon our understanding of brain organisation and function. We offer these as cautionary tales to encourage those who use interference methods in humans to strive for a thorough understanding of how these methods work, lest subtleties in the mechanisms lead to ambiguous or erroneous findings.

### 4.1 What lies beneath: the importance of surgical approach

One notable example of the importance of surgical approach concerns our understanding of the role of the medial temporal lobe (MTL) in memory. The story begins with HM, perhaps the most famous patient in all of neuroscience. Following the removal of both hippocampi, amygdala, as well as significant portions of the MTL, he exhibited profound deficits in anterograde memory; thus introducing the idea that structures within or near the MTL must be involved in memory (Buckley, 2005, Corkin, 2002, Meunier and Barbeau, 2013, Squire and Wixted, 2011).

Later animal lesion experiments appeared to suggest that it was the amygdala and hippocampus that were responsible for recognition memory. In an experiment conducted by Mishkin (1978), monkeys were trained to perform an object recognition task. He found that lesions to one or the other structure (amygdala or hippocampus) produced only mild deficits, whereas the animals that received a combined amygdala-hippocampus lesion exhibited significant deficits in this task. Based on these results, it was argued that the amygdala and hippocampus were responsible for object recognition memory.

However, to lesion the amygdala and hippocampus by aspiration, it was necessary to damage the nearby perirhinal and entorhinal cortices. This raised the possibility that it was not (just) the hippocampus and amygdala, but perhaps nearby cortex and/or fibres of passage that might ultimately be critical for long term memory.

Follow-up experiments revealed this to indeed be the case. Lesions to entorhinal and perirhinal cortices that spared the hippocampus and amygdala showed memory deficits similar to those observed by Mishkin (Meunier et al., 1993b, Murray and Mishkin, 1986). As final confirmation, Murray and Mishkin (1998) later showed that excitotoxic lesions to the amygdala and hippocampus that spared both the rhinal cortex and nearby fibres of passage produced no behavioural deficit.

In this case, the initial results provided by HM suggested a role of hippocampus and amygdala in memory, as these were regions heavily affected by the surgical resection. Ironically, a recent post-mortem examination of HM's brain by Annese et al. (2014) revealed white matter damage in the MTL, as well as spared sections of the amygdala, hippocampus, and ventral temporal regions. Thus, while our knowledge of the role of MTL in memory has greatly increased since the first study by Mishkin and colleagues, it is nonetheless important to recognise that the data upon which they were based were potentially misleading from the start.

More generally, unintentional effects on off-target tissue, whether temporary or permanent, can lead to erroneous conclusions. The long history of lesion methods in animals - particularly ablation methods, which are greatly impacted by ease of surgical access – means that much has been learned about guarding against such off-target effects. This is relevant for interpreting effects of, for example, tDCS in humans, where off-target effects could arise due to actions to the cortex underlying the control electrode, or the route that the current takes to pass between the electrodes.

### 4.2 Collateral damage: the importance of white matter

When creating either a temporary or permanent lesion with some of the techniques discussed, it is difficult to avoid inadvertently affecting nearby white matter. In some cases, this might not have any effect on the experiment or its conclusions. For example, if the white matter is largely targeting the lesioned area. However, in other cases, inadvertent influences on white matter can have significant unintended consequences.

In humans, damage to the orbitofrontal cortex (OFC) produces impulsivity, difficulty in regulating emotions, and other behaviours that are collectively described as ‘acquired sociopathy’ (Bechara et al., 2000; Damasio et al., 1990) – first and most famously described in the case of Phineas Gage (Harlow, 1848). Such lesions also produce difficulty in reversal learning tasks, which require participants to relearn new reward-action associations (Berlin et al., 2004, Jonker et al., 2015, Tsuchida et al., 2010). Aspiration lesions to OFC in monkeys and rats produce altered responses to fear-inducing stimuli (Izquierdo et al., 2005, Rudebeck et al., 2006) as well as difficulty with object-reward associations, assessed with reversal learning tasks (Hornak et al., 2004, Izquierdo, 2004, McAlonan and Brown, 2003, Schoenbaum et al., 2002).

Recently, it was demonstrated that if one makes the lesions using injections of ibotenic acid (and not aspiration), thereby sparing fibres of passage, the effect of damage to the OFC on reversal learning is greatly reduced (Kazama and Bachevalier, 2009, Rudebeck and Murray, 2011). This raised the possibility that the role of OFC in reversal learning and object-reward associations was perhaps overstated and it was in fact damage to neighbouring fibres of passage feeding other parts of the prefrontal cortex that was responsible for these deficits.

Rudebeck et al. (2013) provided an illuminating illustration of this by contrasting injection versus aspiration lesions of the OFC in reversal tasks. Consistent with the aforementioned studies, they found that injection lesions to OFC did not produce any deficit in reversal learning, whereas animals with aspiration lesions showed a significant deficit. They also demonstrated that, unlike monkeys with aspiration lesions to OFC, monkeys with injection lesions to OFC showed similar responses to fear-inducting stimuli as unoperated controls; which raised further questions about the role of OFC in regulating emotions. They did, however, show that both groups of lesioned animals (excitotoxic lesions and aspiration lesions) failed to devalue food choices after a selective satiation procedure, arguing for a role of OFC in value updating.

In summary, these results all suggested that the effects that are typically observed following aspiration lesions to OFC result from damage to fibres of passage, rather than damage to the OFC proper. To confirm this hypothesis, Rudebeck and colleagues conducted a second set of experiments where they used aspiration to lesion a small part of the OFC to specifically target the fibres of passage. These animals showed similar deficits in the emotion regulation and reversal learning tasks as the monkeys who had received complete aspiration lesions to the OFC.

This is a dramatic example of how inadvertently affecting nearby tissue can have drastic effects on our conclusions. More specifically, it also demonstrates how disconnection of distant brain regions due to white matter damage can underlie deficits previously ascribed to nearby cortex [for another example, see Gaffan and Hornak (1997), who showed that parietal cortex lesions only produced neglect in monkeys when the lesion also severed the underlying white matter tract].

### 4.3 Temporary versus permanent lesions

Perhaps the most pertinent cases to consider for researchers seeking to use temporary lesion techniques in humans are those where temporary and permanent lesions in animals have produced different results. An illustrative example of this concerns research examining the role of the frontal eye field (FEF) and superior colliculus (SC) in saccade generation.

The oculomotor circuit is among the most studied and well-understood circuits in the primate brain (Krauzlis et al., 2017, Schall, 1995, Sparks, 2002). It consists of well-characterised anatomical pathways between the eye, visual cortex, prefrontal regions (including the FEF and dorsolateral prefrontal cortex), midbrain superior colliculus, and the premotor circuitry in the brainstem. Of note are the direct connections between the SC and the premotor circuitry, and the direct connections between the FEF and the premotor circuitry. Applying electrical stimulation to either the FEF or SC will evoke short-latency saccadic eye movements (Bruce et al., 1985, Robinson, 1972), which means that either circuit might participate in generating voluntary saccades.

If this is the case, there should be no impairment following damage to either the FEF or SC because the other circuit could theoretically compensate. This was indeed supported by studies using permanent lesions: the animal will experience only transient deficits in saccade generation if one or the other structure is permanently ablated, but persistent deficits if both structures are permanently ablated (Albano et al., 1982, Schiller et al., 1987, Schiller et al., 1980, Schiller and Chou, 1998, Wurtz and Goldberg, 1972). This suggests that the primate brain includes two overlapping but independent pathways for generating saccadic eye movements.

Hanes and Wurtz (2001), found evidence that contradicted this theory. Instead of permanently damaging these areas, they used lidocaine to temporarily inactivate the SC and then electrically stimulated neurons in the FEF. If the two pathways are independent, this procedure should have produced the same result as that observed following permanent lesions to the SC; that is – no effect on saccade generation. However, they found that in many cases they could not evoke saccades through FEF stimulation after temporary inactivation of the SC. In those cases where they were able to evoke saccades (approximately 50% of cases), there was abnormal variability in saccadic endpoint. The interpretation here is that following a permanent lesion to one structure, the other structure can compensate through reorganisation – but only after a delay.

This example of contrasting effects of permanent versus temporary lesions should provide additional incentive for the use of temporary methods in both humans and animals. Of the two, temporary lesion methods provide the best opportunity to examine the *immediate* consequences of removing a given brain region; that is – prior to any significant network reorganisation or the acquisition of any compensatory behavioural strategies.

And yet, it is vital not to ignore the possibility of off-target consequences of inactivating an area. We have already discussed consequences of inadvertently but *directly* affecting brain regions as a result of the experimental intervention. There is also, however, the possibility of *indirectly* affecting the function of a downstream region by temporarily disrupting its input. This phenomenon was recently demonstrated by Otchy and colleagues, in an elegant experiment involving songbirds (Otchy et al., 2015). We include a brief summary of this experiment.

In the zebra finch, song production involves the function of (at least) two interconnected regions: the nucleus interface (Nif) and the HVC (a.k.a., the High Vocal Centre). Permanent lesions of Nif do not disrupt song production, whereas temporary lesions do. One possibility, therefore, is that Nif is indeed necessary for song production but that compensatory mechanisms can be recruited to eventually overcome its loss.

To test this theory, Otchy and colleagues first temporarily inactivated Nif while simultaneously recording neural activity from HVC. They observed that the deficit in song production following temporary inactivation of Nif closely resembled that observed following permanent lesions to HVC. They also demonstrated that temporarily disrupting Nif resulted in altered neural activity in HVC. In follow-up experiments, they repeated a similar procedure, this time permanently lesioning Nif while recording neural activity in HVC. Immediately after the lesion, they once again observed disrupted song production and altered neural activity in HVC. Critically, however, over 1–2 days following the lesion song production gradually recovered and neural activity in HVC returned to normal prelesion levels.

Otchy et al. (2015) were thus able to show that it was not removal of Nif that was directly responsible for disrupted song production, but rather the off-target effect on neural activity in HVC. Such findings are not only a reminder to consider off-target effects of temporary or permanent lesions, but are also an example of how animal lesion research can enable exquisite insights into brain networks and recovery processes.

## 5 How can animal studies complement human studies?

Now that reversible approaches are emerging in human research, does this mean that lesion studies in animals will no longer be necessary? This is a question that one of us (AB) is often asked. As explained in the previous section, having a thorough understanding of how a given technique works is essential for drawing correct conclusions from the data. This is one area where the use of animal models can directly benefit those seeking to use reversible techniques in humans. We argue that our goal as a scientific community should be to gain a complete understanding of how techniques such as TMS or tDCS affect neuronal activity, both in the target site and in any neighbouring tissue. This will inevitably require that we combine these techniques with measurements of cellular activity, necessitating methods that are only possible in animals (at least for the foreseeable future).

For example, recent studies have combined TMS with invasive recordings in monkeys to reveal important insights about how TMS influences neuronal activity (e.g., Mueller et al., 2014; Papazachariadis et al., 2014). Another study used PET scanning in anaesthetised monkeys to examine how dopamine release is affected by repetitive TMS (e.g., Ohnishi et al., 2004). Similar combinations will undoubtedly continue to reveal vital insights as to the effects of the tools we seek to use in humans.

## 6 Conclusions

For the study of most cognitive functions, lesion studies in animals have and will likely continue to provide insights that cannot be obtained through research on humans. In this review, we have provided a primer on permanent and reversible lesion techniques currently in use in animal research, and a brief discussion of how they might complement and extend human neuropsychological research. With the tools available in animals, neuropsychology can address not just the removal of representations and disconnection effects caused by lesions, but also questions about *how* a brain area contributes to an aspect of cognition. Finally, research in animals will continue to clarify the precise mechanisms underlying methods used in human research, such as the neuronal and pharmacological consequences of temporary interference methods like TMS, or the time course and nature of reorganisation following permanent lesions.
